# Plasma obtained following murine hindlimb ischemic conditioning protects against oxidative stress in zebrafish models through activation of nrf2a and downregulation of duox

**DOI:** 10.1371/journal.pone.0260442

**Published:** 2021-11-24

**Authors:** Rui Guan, Xiao-yan Wen, Chung Ho Leung, Caterina Di Ciano-Oliveira, Stephen Lam, Si Yuan Dai, Farhad Karbassi, Antonio Mauro, Youdong Wang, Ori Rotstein

**Affiliations:** 1 Zebrafish Centre for Advanced Drug Discovery, The Keenan Research Centre for Biomedical Science Toronto, Li Ka Shing Knowledge Institute, St. Michael’s Hospital, Ontario, Canada; 2 Department of Medicine & Institute of Medical Science, University of Toronto, Toronto, Ontario, Canada; 3 Departments of Surgery, St. Michael’s Hospital and University of Toronto, Toronto, Ontario, Canada; Washington State University, UNITED STATES

## Abstract

Ischemia/reperfusion of organ systems in trauma patients with resuscitated hemorrhagic shock (HSR) contributes to tissue injury and organ dysfunction. Previous studies using a murine model of HSR showed that remote ischemic preconditioning (RIC) protected against organ injury and that the plasma was able to prevent neutrophil migration in a zebrafish tailfin-cut inflammation model. In this study, we hypothesized that RIC plasma inhibits neutrophil function through a decrease in reactive oxygen species (ROS) production via the upregulation of the transcription factor Nrf2 and downstream antioxidative genes. Plasma from mice subjected to RIC (4 cycles of 5-min hindlimb ischemia/reperfusion) was microinjected into zebrafish. The results show that RIC plasma caused a reduction of ROS generation in response to tail injury. In addition, RIC plasma protected the fish larvae in the survival studies when exposed to either H2O2 or LPS. Oxidative stress PCR Array showed that RIC plasma treatment led to upregulation of antioxidative related genes including hsp70, hmox1a, nqo1 as well as downregulation of duox, the producer of H2O2. To explore the role of nrf2 in RIC, RIC plasma from Nrf2 KO mice were injected to the zebrafish and showed no inhibitory effect on neutrophil migration. Moreover, knockdown of nrf2a attenuated the anti-inflammatory and protective effect of RIC plasma. The downregulation of duox and upregulation of hmox1a were confirmed to require the activation of nrf2a. Therefore, we show that the protective effect of RIC may be related to the elaboration of humoral factors which counter injury-induced ROS generation in a nrf2-dependent fashion.

## Introduction

Cellular injury resulting from sequential ischemia/reperfusion (I/R) of tissues is a pathophysiological process which contributes to morbidity and mortality in a wide range of medical settings, including ischemic stroke, myocardial infarction, organ transplantation and trauma-induced hemorrhagic shock [1]. Typically, the balance between metabolic supply and demand is altered due to hypoxia within the ischemic organ and subsequent reperfusion further exacerbates the metabolic imbalance by enhancing the activation of immune responses and a cascade of inflammatory processes [2, 3]. While a variety of molecular mechanisms have been proposed to explain this process, excess generation of reactive oxygen species (ROS) and infiltration of neutrophils are considered to be critical factors in the pathogenesis of I/R injury [2]. Neutrophils in the circulation perform an essential host-defense function by directly migrating to the site of injury for tissue repair, but excess neutrophils may cause tissue damage, in part through the generation of oxidative stress [4]. ROS are able to directly injure cells, but may also promote pro-inflammatory signaling cascades which exacerbate injury [2, 5, 6]. As a part of the host response, oxidative stress is known to induce a broad antioxidant response through the generation of neutralizing antioxidant proteins. The transcription factor nuclear factor erythroid 2-related factor (nrf2) is a known regulator of the antioxidant response, by virtue of its ability to induce antioxidant response element-dependent genes.

In response to I/R, especially due to hypoxia, more ROS is produced through electron leakage from mitochondria electron transportation chain and activation of NADPH oxidase. The major mechanism by which cells control their antioxidant response is mediated through activating the transcription factor nuclear factor nrf2 [7–9]. Nrf2 can bind to the antioxidative responsive elements (ARE) in the promoter region of antioxidative genes and initiate their transcription expression. Under normal conditions, Nrf2 binds to Kelch-like ECH-associated protein 1 (keap1), which promotes ubiquitination of Nrf2 and eventual degradation of Nrf2 in cytosol. Under oxidative stresses, cysteine residues in Keap1 enable it to act as a redox sensor, detecting changes in cellular redox state [8, 10]. The oxidation of cysteines residues (C151, C273, C288, C613) inactivates Keap1 and results in the stabilization and translocation of Nrf2 to nucleus. In the nucleus, Nrf2 heterodimerizes with small protein MAF and binds AREs, and activates detoxifying enzymes and antioxidant enzyme [11, 12].

Zebrafish have been used as a model organism to study vertebrate development and disease due to its genetic similarity to the human genome, rapid embryonic development, high fecundity, ease of genetic manipulation and transparent embryos which facilitate fluorescence imaging. Larval zebrafish have been frequently used to study myeloid cell function [13, 14]. Zebrafish neutrophils are identifiable from approximately 48 hours post fertilization (hpf) and show the same morphological, biochemical and functional features as mammalian neutrophils. They have a polymorphic nucleus, primary and secondary granules, myeloid-specific peroxidase and a functional NADPH oxidase [15]. Moreover, larval zebrafish provide a time window to focus on innate immunity and neutrophil studies, independent of adaptive immune responses, which are functionally mature 4 to 6 weeks after fertilization. Transgenic zebrafish with neutrophils specifically labeled with fluorescent proteins have previously been generated [13, 16]. These models facilitate excellent real-time visualization of neutrophil migration in vivo toward tissue damage and have led to the adoption of zebrafish as a model organisms for in vivo neutrophil study [15, 17, 18]. Application of these models has underpinned many key advances in the understanding of inflammation, including the identification of a tissue scale gradient of hydrogen peroxide for neutrophil recruitment in response to tissue injury [6, 19, 20].

Remote ischemic conditioning (RIC) is a therapeutic intervention shown to prevent the onset of organ I/R injury in both experimental animal models and also human trials [21, 22]. This procedure is performed by applying brief repetitive cycles of ischemia/reperfusion to a limb. Using a murine model of hemorrhagic shock/resuscitation (S/R), our previous study demonstrated that RIC exerted a protective effect on S/R-induced liver and lung injury [21]. The protective effect of RIC was associated with the liberation of humoral factors that exerted anti-inflammatory effects on neutrophil function. However, the mechanism underlying this anti-inflammatory effect in zebrafish is currently unknown. The ability to transfer protection using RIC plasma coupled with the ease of studying inflammation in zebrafish larvae together represent an attractive experimental model for addressing potential mechanisms. Given the role of oxidative stress in propagating inflammation in this model, we hypothesized that RIC may exert protection through upregulating antioxidant response to protect against I/R injury.

## Materials and methods

### Zebrafish care and general procedure

Zebrafish strains TU, Tg(mpx:GFP) (male and female, 6 months old) were raised and maintained using standard laboratory procedures as described [23]. Embryos were obtained via natural mating and cultured in embryo E2 buffer. All experiments in this study were conducted according to the ethical guidelines established by the St. Michael’s Hospital Animal Care Committee and Research Ethics Board with approved animal protocol ACC#660 and #867. All researchers worked with zebrafish were trained by zebrafish facility and vivarium facility.

Studies in this paper are completed on larval zebrafish between 3–14 days post-fertilization (dpf). Euthanasia of zebrafish at different stages are carried out by the following methods in accordance with the AVMA guidelines on Euthanasia.

For zebrafish ≥8 dpf, rapid chilling is used for euthanasia by submersion in ice water (0–4º C) for at least 10 minutes following cessation of opercular (i.e., gill) movement. In any fish where it is difficult to visualize opercular movement, fish should be left in the ice water for at least 20 minutes after cessation of all movement to ensure death by hypoxia. For zebrafish 4–7dpf, beaching is used for euthanasia by adding bleach solution (sodium hypochlorite 6.15%) to the culture system water at 1 part bleach to 5 parts of water. The fish larvae were kept in this solution at least five minutes to ensure death. Pain perception has not developed at these earlier stages, so this is not considered a painful procedure.

For embryos ≤ 3dpf, the development of embryos is terminated using bleach solution (sodium hypochlorite 6.15%) for at least five minutes.

### Remote ischemic conditioning

Male mice (C57BL/6 or Nrf2 Knockout, 9–11 weeks old) were subjected to RIC by 4 cycles of 5-min hindlimb occlusion/release or anesthesia only (Control). Blood was immediately collected after RIC into citrated tube and spun down to acquire plasma. The mice were anesthetized with isoflurane (3% for induction and 2% for maintenance). After the surgery, all the mice were euthanized with 5% isoflurane.

### Zebrafish microinjection

RIC plasma or control plasma were obtained and diluted 1:20 with saline for zebrafish embryo injection. This 5% dilution was based on pilot studies showing that this was the optimal dilution, with 1% showing no effect and 10% showing some toxicity. Three days post fertilization (dpf) Tg(mpx:GFP) larvae were anesthetized by immersion in clove oil. A volume of 2 nl plasma or vehicle (saline) were microinjected into the common cardinal vein of zebrafish larvae at 3dpf. Larvae were then incubated for 16 hours.

### Tailfin transection

Larvae were anesthetized in E2 buffer containing 0.1 mg/ml Tricaine prior to wounding. Tailfin transection was performed with a 30-gauge needle, which is sterilized using 70% ethanol prior to use. A single cut was made traversing the entire dorsoventral length of the caudal fin, posterior to muscle and notochord in each fish. Larvae were incubated for 3 hours at 32C and then imaged using fluorescence stereomicroscopy.

### nrf-2a and nrf2b morpholino knockdown in zebrafish

The zebrafish nrf2 morpholinos (MOs) was previously described [24]. nrf2a-MO: (5’-CATTTCAATCTCCATCATGTCTCAG-3’); nrf2b-MO: (5’-AGCTGAAAGGTCGTCCATGTCTTCC-3’). nrf2a or nrf2b MO was designed to block the ATG start site of its mRNA. The standard control-MO from Gene Tools (Oregon, USA) was also used (5’-CCTCTTACCTCAGTTACAA TTTATA-3’). For the morpholino injection, 2 nl of 0.2mM nrf2a-MO, or nrf2b-MO or standard control-MO were injected into Tg(mpx:EGFP) fish embryos at one-cell stage, respectively. To evaluate the effect of RIC serum on nrf2-MO fish, RIC or Ctrl plasma are injected into the common cardinal vein of 3 dpf larvae which had been injected with morpholino.

### Oxidative stress RT² profiler PCR array

Zebrafish larvae at 3dpf were injected with Ctrl or RIC plasma and incubated at 28°C for 16 hours. Larvae were immediately placed in Trizol and stored at −80°C until total RNA isolation and gene expression analysis. Total RNA was prepared by phenol-chloroform extraction method (TRIzol, Thermo Fisher) and DNA was eliminated by using DNase digestion from a RNA purification kit (QIAGEN). After that RNA was further purified by RNeasy MiniElute Clean Up Kit (QIAGEN). RT2 First Strand Kit was used to synthesize cDNAs and to eliminate genomic DNA. Zebrafish Oxidative Stress RT² Profiler PCR Array was performed according to the manufacturer’s protocol. Data were analyzed by SABioscience’s web-based PCR array analysis software using the comparative Ct method with the formula (2-DDCt). A set of housekeeping genes (acta1b, b2m, hprt1, nono, rpl13a) were used for normalization.

### Zebrafish H2O2 and LPS exposure

RIC plasma-injected, Ctrl plasma-injected or Saline-injected larvae were exposed to H2O2(25mM) or LPS (80 μg/mL and 100 μg/mL) at 28°C. The chemical exposure started at 16 hours after microinjection in triplicates in 12-well plastic dishes. Each well contains 25 larvae in 2.5 ml of 0.3× Danieau’s solution. Following exposure, tail edema, mortality and survival were assessed in the following days. The evaluation of motility was identified with ventricular asystole. Non-injected controls were included on every plate.

For the survival studies, the humane endpoint is defined by asystole (the cessation of the heartbeat) and the cessation of the body movements (no touch response). LPS and H2O2 causes death in zebrafish by tissue damage and severe inflammation. The survival studies for LPS treatments lasted for 24 hours (high concentration) and 8 days (low concentration), respectively. The fish larvae treated with LPS at the concentration of 100μg/mL were observed and checked every 4 hours throughout the treatment. The fish larvae treated with LPS at the concentration of 80μg/mL were monitored every 6 hours. The fish larvae showing asystole and no movements (no touch response) was recorded. Immediately they were removed from the plate and submerged in ice water for at least 20 minutes. For this experiment, there were no fish larvae died before meeting criteria for euthanasia. By the end of the experiment, all the fish larvae which remained alive were euthanized by submersion in ice water or bleach solution according to their ages as described in the euthanasia section.

### ROS generation assay by CellROX

Tailfin transections were performed on zebrafish larvae at 4dpf. The CellROX Reagent (Thermo fisher) was added to the fish water at a final concentration of 5 μM and incubated for 30 minutes at 32°C. Fish water with CellROX was removed and the larvae were rinsed 3 times with clean fish water. Live imaging of larva was performed using a Zeiss Axiovert wide field microscope system, images were taken every 10 minutes after tail fin transection. The background subtracted integrated fluorescence intensity of CellROX in the tailfin region was then quantified using Zen software.

### Real-time PCR

RNA purification and cDNA synthesis were performed as described above. Real-time PCR was performed using RT2 SYBR Green qPCR master mixes (SABiosciences, Hilden, Germany) to determine the relative cDNA levels of duox and hmox1a, in each sample. The sequences of primers are as following: duox-fwd: 5’-GTT GGC TTT GGT GTA ACT GTA-3’; duox-rev: 5’-GCC CAG GCT GTG AGA G-3’; hmox1-fwd: 5’-GCTTCTGCTGTGCTCTCTATACG-3’; hmox1-rev: 5’-CCAATCTCTCTCAGTCTCTGTGC-3’; β-actin-fwd:5’-CAACAGAGAGAAGATGACACAGATCA-3’; β-actin-rev:5’-GTCACACCATCACCAGAGTCCATCAC-3’; ef1a-fwd:5’-CTTCTCAGGCTGACTGTGC-3’; ef1a-rev: 5’-CCGCTAGGATTACCCTCC-3’. β-actin and ef1a were chosen as the housekeeping gene for normalization. Threshold cycle (Ct) values were collected and used for ΔΔCt analysis. Experiments were performed in triplicate and repeated at least three times.

### Statistical analysis

Data are expressed as mean ± standard deviation. Comparisons between multiple groups were performed by ANOVA followed by Tukey’s post hoc test. Pairwise comparisons were performed by t-test. P-value of < 0.05 was considered significant. Comparison between groups in zebrafish survival studies were performed by log-rank Mantel-Cox test. All experiments were performed at least three times.

## Results

### RIC plasma reduced neutrophil migration and ROS generation at the site of injury

**Fig 1:**
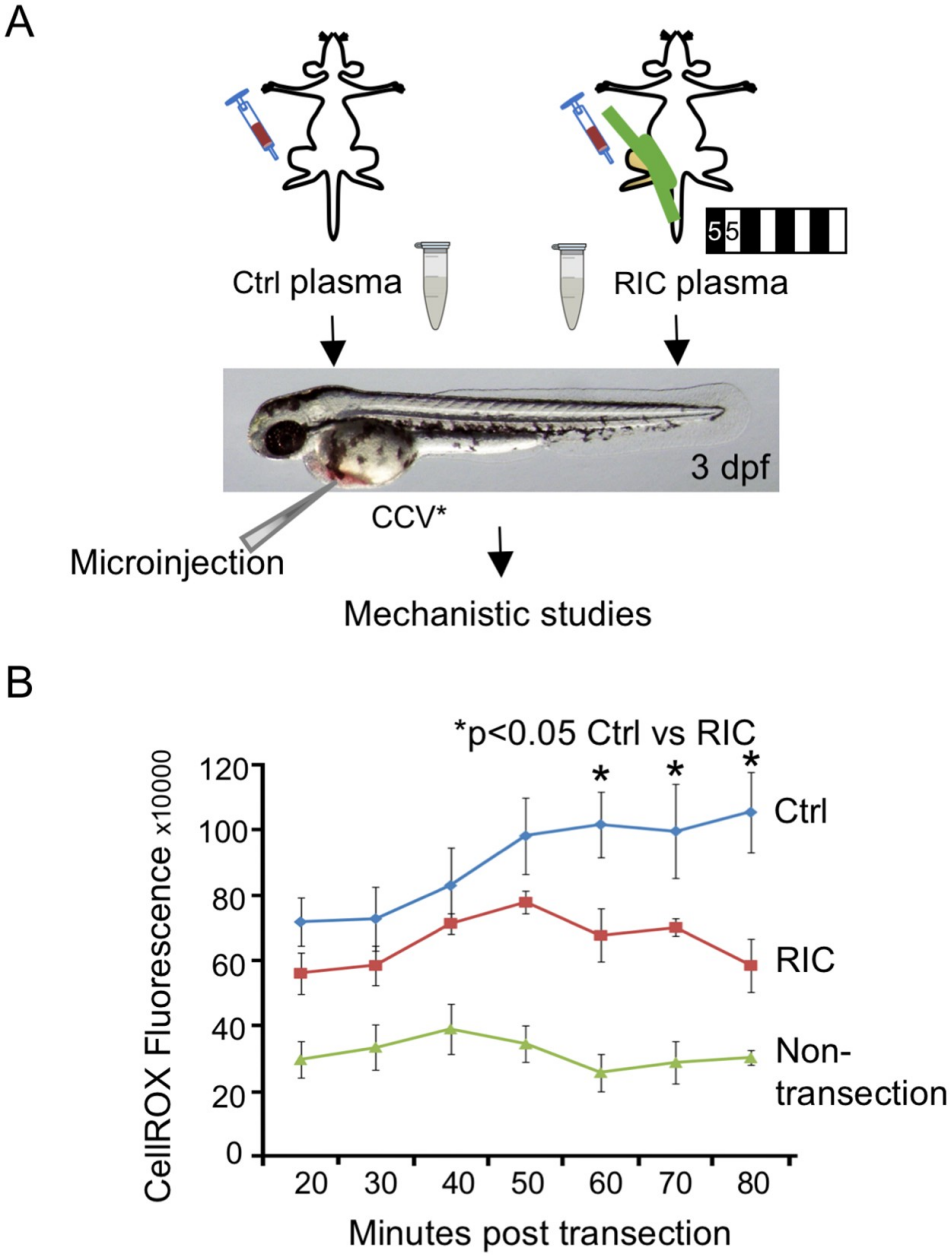
RIC plasma reduces ROS generation in the tail wound in zebrafish. (A)Plasma from mice subjected to RIC (4 cycles of 5-min hind limb ischemia/reperfusion) or control (Ctrl) was microinjected into common cardinal vein of Tg (mpx:EGFP) zebrafish at 3 day post fertilization. (B) The graph shows the CellROX fluorescence intensity detected at different time points in RIC, Ctrl and non-tailfin transection groups. *p<0.05.

We previously reported [21] that RIC plasma from mice reduced the neutrophil migration to the tail wounds compared to control (Ctrl) plasma and saline injected fish. In the current manuscript, we performed the procedures as shown in Fig 1A and carried out neutrophil migration studies again to provide context for the current work. As shown in S1 Fig, there were significantly fewer neutrophils recruited in the tail wounding area in RIC plasma injected fish, compared to both saline and Ctrl plasma at 1.5 hours and 4.5 hours post tail transection (p<0.001). In order to exclude the possibility that the decrease of neutrophils in the tail wound by RIC was due to the decrease of neutrophils in the whole fish body, we counted total body neutrophils and found that there was no significant difference in neutrophil counts between saline (237 ± 29, n = 12), Ctrl (229 ± 12, n = 12) and RIC plasma injected fish larvae (233 ± 20, n = 12)(S2 Fig), suggesting that RIC causes reduction of neutrophil migration to the tail wound without affecting the total number of neutrophils in the fish. This anti-inflammatory response with RIC plasma suggested that RIC may induce factor(s) in the circulation which directly or indirectly generate an effect in the zebrafish that mitigates neutrophil migration into cut region of the tailfin cut.

Work by Niethammer and colleagues [20] demonstrated that the directional migration and tissue infiltration of neutrophils are mediated by H2O2 gradient, we further investigated whether RIC might exert an effect on the level of ROS generation at the tail transection region. As shown in Fig 1B and S3 Fig Fiby CellROX assay, there was a time-dependent increase in ROS generation in Ctrl plasma injected fish larvae. This rise was significantly reduced in RIC plasma-injected fish larvae. This finding suggests that RIC could reduce ROS generation at the site of injury through humoral factors in the plasma.

### RIC plasma improves survival in zebrafish exposure to induced-oxidative stress

Having shown that RIC plasma was able to inhibit ROS generation, and this correlated with impair neutrophil migration, we asked whether RIC plasma might impact on other ROS-induced inflammatory processes.

**Fig 2:**
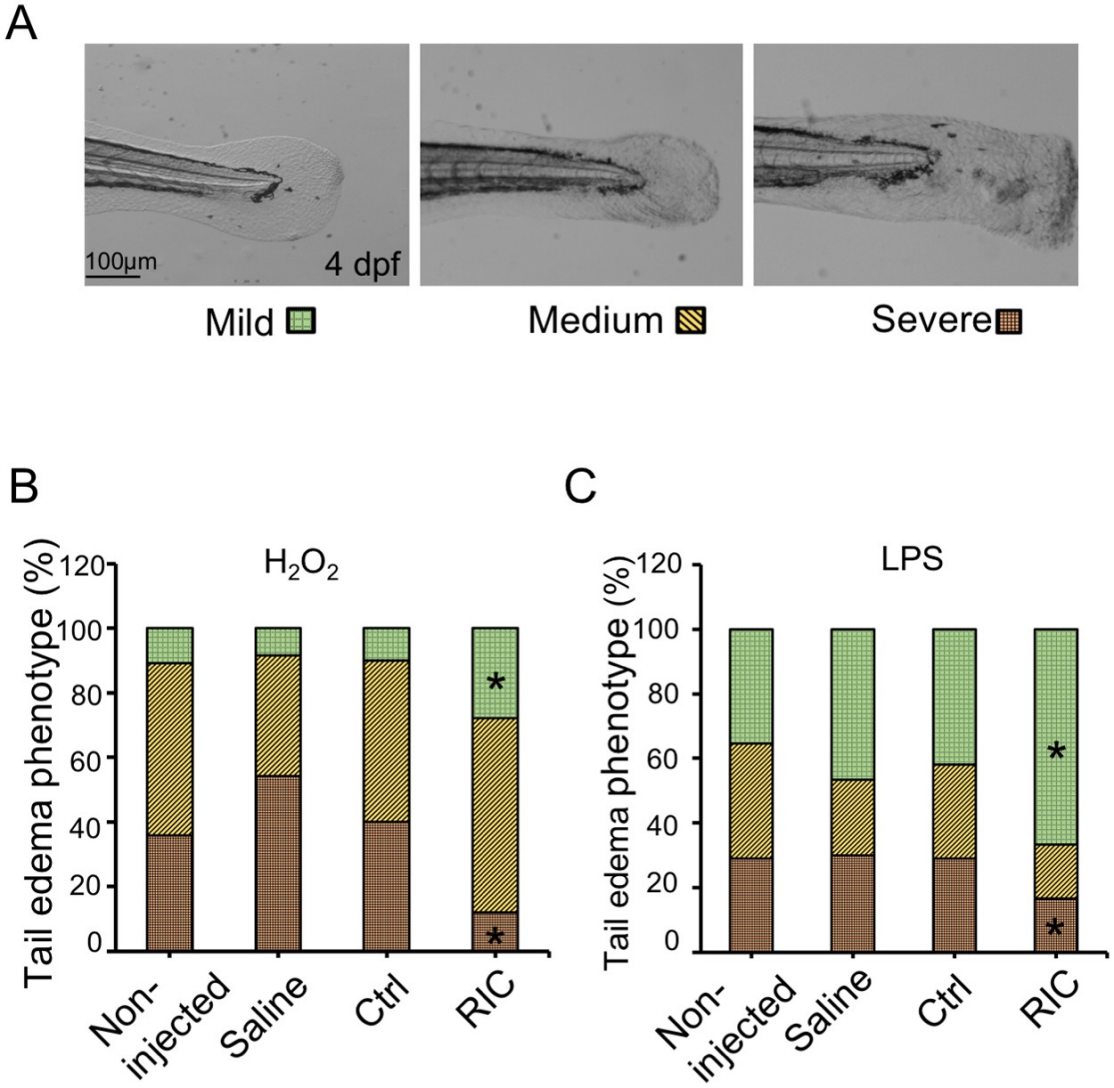
RIC protects against oxidative stress induced by H2O2 or LPS. (A) H2O2 or LPS induces tail fin edema in zebrafish larvae. The phenotype of the tailfin edema was categorized into three groups according to the extent of damage: mild, medium, and severe. (B) Zebrafish injected with saline, Ctrl or RIC Plasma were subjected to H2O2 (25mM) for 1.5 hours. Percentage of each phenotype for each group were shown in graph. *P < 0.05. (C) zebrafish injected with saline, Ctrl or RIC Plasma were subjected to LPS (100 μg/mL) for 6 hours. Percentage of each phenotype for each group were shown in graph. *P < 0.05.

In zebrafish, oxidant-mediated inflammation leading to tailfin edema and mortality can be studied by exposing embryos to exogenous hydrogen peroxide (H2O2) for sterile inflammation or lipopolysaccharide (LPS) to mimic infectious inflammation. As shown in Fig 2A, H2O2 or LPS exposure caused tailfin damage and edema in larval zebrafish. We categorized the phenotype of the tailfin edema into three groups: mild, medium, and severe. For mild edema, the tailfin edges stay in the round shape with skin intact and minor edema; for medium edema, the tailfins are swollen; and for the severe edema, viscus exudate accumulated in the tip of the tailfin and elongated its shape. To evaluate whether RIC plasma could protect the larvae against oxidant-induced edema, zebrafish at 3 days post-fertilization (dpf) were microinjected with Ctrl or RIC plasma and exposed to H2O2 (25 mM for 90 minutes). Zebrafish larvae treated with RIC plasma showed a significantly lower percentage of severe phenotype (12% vs 40%), and a significantly higher percentage of mild edema phenotype (28% vs 10%) compared to the fish treated with Ctrl plasma as illustrated in Fig 2B, implying that RIC attenuates the severe tissue damage from H2O2 exposure. We also examined the effect of LPS which induces and mediates inflammation by increasing ROS generation [25]. Zebrafish exposed to LPS following treatment with RIC plasma demonstrated a lower percentage of severe tailfin edema (17% vs 29%), and a higher percentage of mild phenotype (67% vs 42%) compared to the Ctrl plasma treated fish (Fig 2C).

We also performed survival studies to explore the role of RIC in response to LPS exposure. When subjected to LPS at 100 μg/mL as shown in Table 1A, zebrafish larvae treated with RIC displayed higher survival (56%) compared to larvae with Ctrl (40%) at 16-hours (p<0.01) and also at the 24-hour time point (27% vs 0%, RIC vs Ctrl respectively, n = 40, p<0.01). We also used a lower dose of LPS (80μg/ml) to examine a less toxic dose and again, animals injected with RIC plasma showed significantly higher survival by day 6 dpf and beyond compared to fish larvae injected with Ctrl plasma (Table 1B) (p<0.01). The details of the number of fish larvae (alive/dead/total) are shown in Table 1.

**Table 1:** Survival of RIC plasma-injected fish compared with control plasma-injected fish after treatment with LPS.

**A. Treatment**	**Survival rate with LPS (100ug/mL)**			
	4–12hour	16hour	20hour	24hour
**Saline**	100%	35%(11/20/31)	29%(9/22/31)	3%(1/30/31)
**Ctrl plasma**	100%	40%(13/19/32)	25%(8/24/32)	0%(0/32/32)
**RIC plasma** **	100%	56%(17/13/30)	40%(12/18/30)	27%(8/22/30)
**B. Treatment**	**Survival rate with LPS (80ug/mL)**			
	1–5day	6day	7day	8day
**Saline**	100%	12%(5/37/42)	5%(2/40/42)	0%(0/42/42)
**Ctrl plasma**	100%	53%(24/21/45)	4%(2/43/45)	0%(0/45/45)
**RIC plasma** **	100%	93%(41/3/44)	52%(23/21/44)	29%(13/31/44)

**Survival rates between saline, ctrl and RIC groups were performed by log-rank Mantel-Cox test. In both A and B, RIC plasma injected fish showed higher survival compared to control plasma injected fish, **p<0.01.

### PCR array displays differential gene expression involved in oxidative stress, antioxidant defense, and reactive oxygen metabolism in RIC treated zebrafish

**Fig 3:**
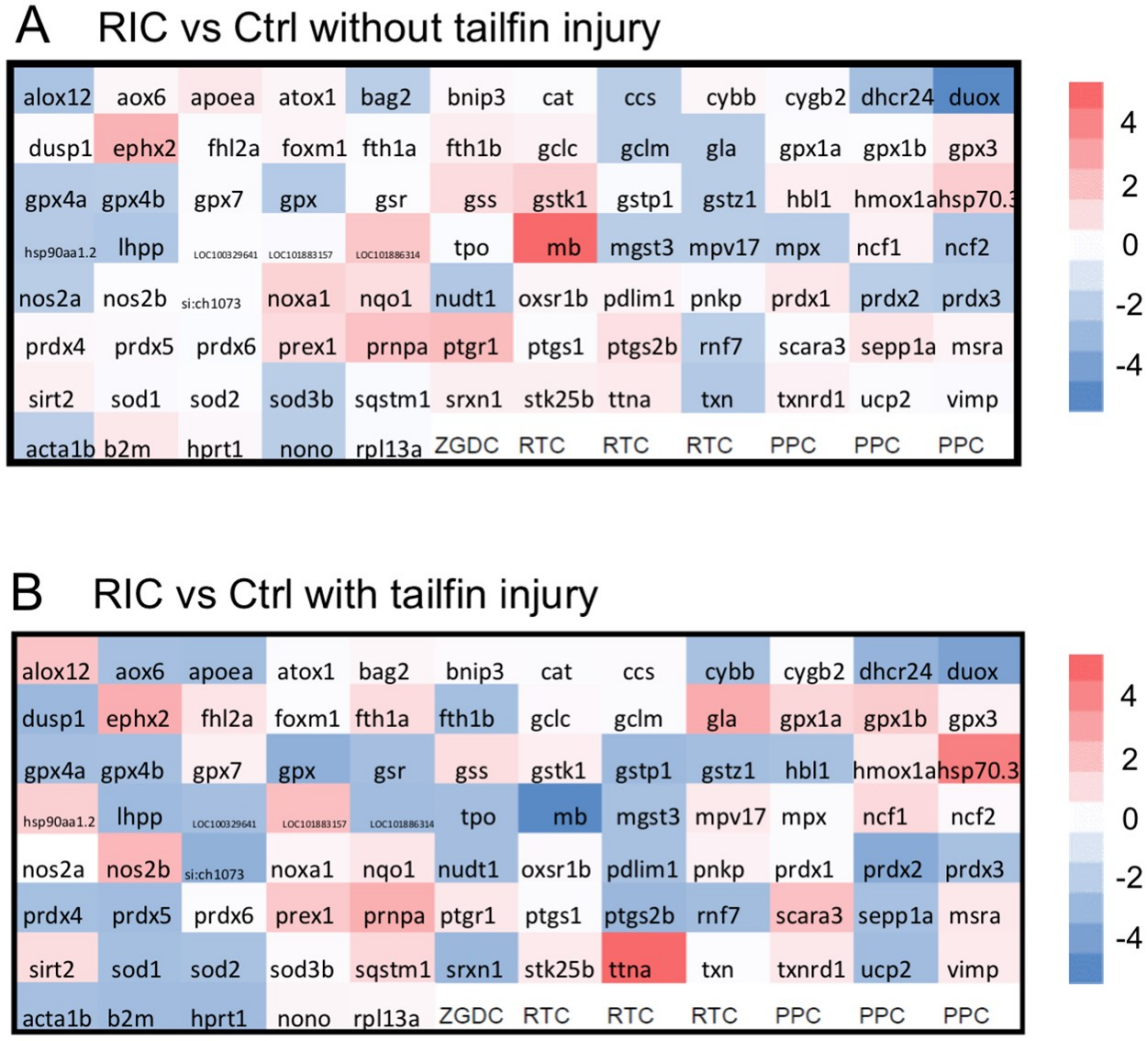
Real-time PCR array revealed that oxidative stress related genes are affected in RIC treated fish. (A) Zebrafish larvae injected with Ctrl or RIC plasma were incubated at 28°C for the same amount of time as the tailfin cut group was harvested. The fish larvae without tailfin injury were collected for RNA extraction and PCR array analysis. The relative gene expression (fold change, RIC group/ Ctrl group) is shown in the heatmap. The up-regulated genes in RIC treated fish are represented in red (fold>1), while the down-regulated gene are represented in blue (fold<-1). (B) Zebrafish larvae injected with Ctrl or RIC plasma were incubated at 28°C for 16 hours. The tail fin was transected and incubated for 1 hour. The fish larvae with tailfin injury were collected for RNA extraction and PCR array analysis.

**Fig 4:**
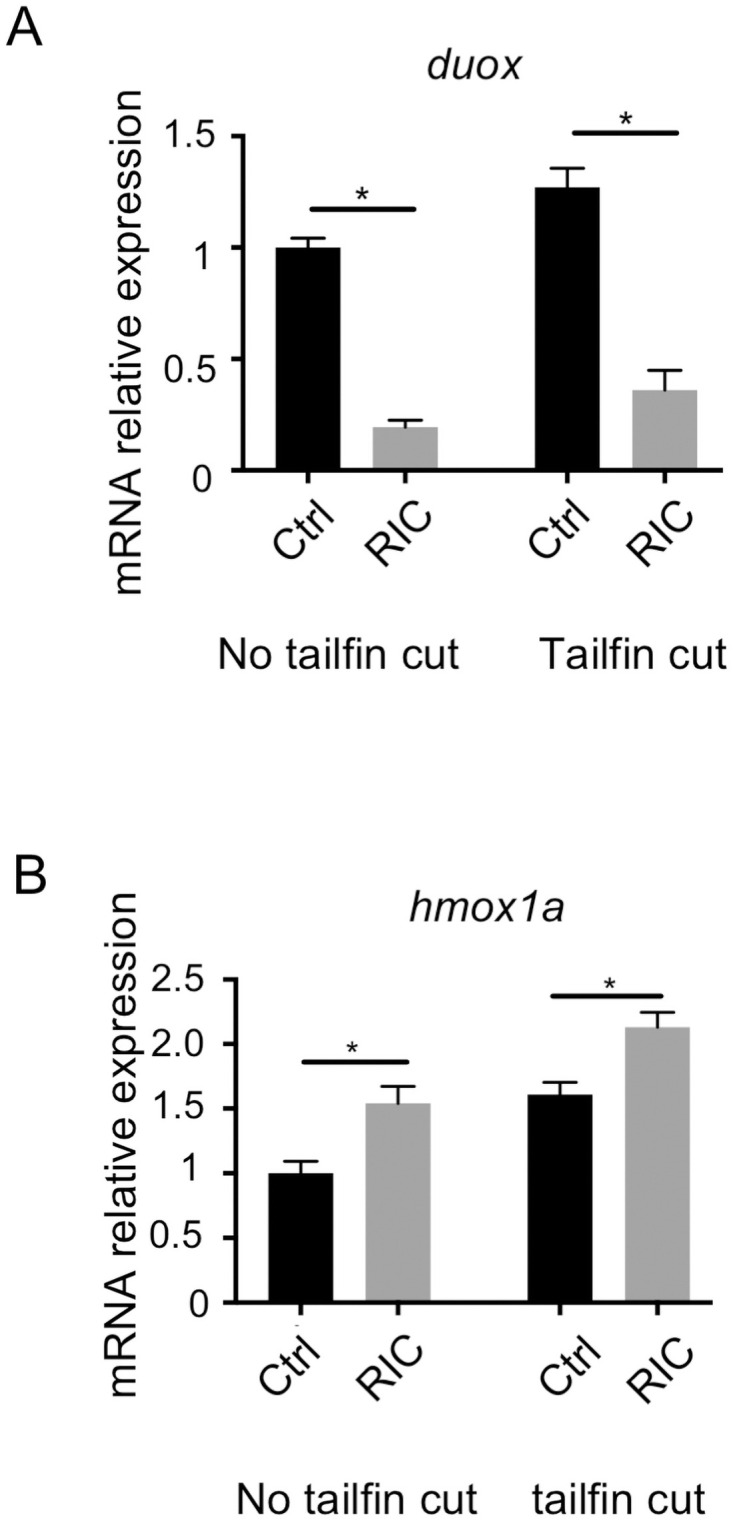
The downregulation of duox and upregulation of hmox1a are confirmed in RIC-treated fish. (A) Real-time PCR data confirmed that the expression of duox at mRNA level is significantly down-regulated in RIC treated fish (p<0.05). (B) Real-time PCR analysis confirmed that the expression of hmox1a at mRNA level is significantly up-regulated in RIC treated fish (p<0.05).

Injurious stimuli including LPS and the tailfin cut have been shown to induce toxicity/inflammation via induction of oxidative stress [20, 25]. The improved survivability of fish following LPS and the lesser migration of neutrophils to the tailfin cut following RIC treatment, which correlated with a reduction of oxidative stress in the zebrafish (Fig 1B), together suggested that RIC might exert its protective role via reduction in oxidative stress. To explore this as a potential mechanism, we studied the effect of RIC on oxidative stress genes using the zebrafish Oxidative Stress PCR Array. This PCR Array profiles 84 related genes simultaneously by real-time PCR with highly reliability and sensitivity. The targeting genes in the array include antioxidants, ROS metabolism related genes, oxidative responsive genes and oxygen transporters. Comparison of gene expression at the mRNA level between RIC injected and the Ctrl plasma injected zebrafish was performed under two conditions: fish larvae with tailfin transection or without tailfin transection. The relative expression of target genes is displayed in heatmaps as Fig 3A and 3B, which displayed the transcriptional profiles of genes involved in oxidative stress, antioxidant defense, and reactive oxygen metabolism. In these two heatmaps, differential expression of the 84 genes was represented by color: upregulated in red, downregulated in blue. Genes were considered to be expressed differentially in RIC plasma treated zebrafish as compared to Ctrl plasma if the changes are more than two-fold. The detailed transcriptional profiling of all the oxidative stress related genes is shown in Table 2. We found that in 95% (80/84) of genes, the expression was changed less than two-fold; Four genes were differentially expressed over two-fold (Table 3). In the absence of tailfin cut, the three upregulated genes were titin (ttna), heat shock protein 70 (hsp70) and myoglobin (mb), while duox was markedly downregulated. Following tailfin cut, mb changed from being upregulated to being downregulated by RIC. A description of function is included in the table, with DUOX and MB having potential impact on ROS production. Real-time PCR confirmed its downregulation in Fig 4A. This suggests that downregulation of duox, the hydrogen peroxide producer, may contribute to the reduction of ROS generation for the RIC treated fish.

**Table 2:** mRNA relative level (fold change) in RIC plasma-injected fish compared to Ctrl plasma injected fish.

Gene name	fold change (up-regulation or down-regulation)	
	intact fish larvae	fish larvae with tail fin cut
**Antioxidants Glutathione peroxidases**		
Glutathione peroxidase 1a (Gpx1a)	1.05	1.36
Glutathione peroxidase 1b (Gpx1b)	1.10	1.40
Glutathione peroxidase 3 (Gpx3)	1.30	1.12
Glutathione peroxidase 4a (Gpx4a)	-1.17	-1.11
Glutathione peroxidase 4b (Gpx4b)	-1.09	-1.08
Glutathione peroxidase 7 (Gpx7)	1.06	1.13
Glutathione peroxidase 8 (Gpx8)	-1.23	-1.65
Glutathione S-transferase kappa 1(Gstk1)	1.59	1.15
Glutathione reductase (Gsr)	1.08	-1.10
Glutathione synthetase (Gss)	1.43	1.29
Glutathione S-transferase pi 1(Gstp1)	1.00	-1.31
Zgc:92869 (gstz1)	-1.06	-1.16
**Peroxiredoxins**		
Peroxiredoxin 1 (Prdx1)	1.30	1.02
Peroxiredoxin 2 (Prdx2)	-1.04	-1.85
Peroxiredoxin 3 (Prdx3)	-1.18	-1.04
Peroxiredoxin 4 (Prdx4)	1.14	-1.10
Peroxiredoxin 5 (Prdx5)	1.12	-1.22
Peroxiredoxin 6 (Prdx6)	1.03	1.04
**Other peroxidases**		
Catalase (Cat)	1.07	1.02
Cytochrome b-245, beta polypeptide (chronic granulomatous disease) cybb,	1.15	-1.06
Cytoglobin 2 (cygb2)	1.03	1.02
Zgc:56518 (mgst3)	-1.10	-1.05
Myeloperoxidase (Mpx)	-1.10	1.03
Prostaglandin-endoperoxide synthase 1 (Ptgs1)	1.07	1.04
Prostaglandin-endoperoxide synthase 2b (Ptgs2b, cox2)	1.39	-1.39
Titin a (Ttna)	1.28	2.22
**Other antioxidants**		
Sulfiredoxin 1 homolog (Srxn1)	1.23	1.39
Thioredoxin reductase 1 (Txnrd1)	1.21	1.20
Superoxide dismutases 1 (Sod1)	1.02	1.13
Superoxide dismutases 2 (Sod2)	1.09	-1.01
Superoxide dismutases 3, extracellular b (Sod3b)	-1.03	1.06
Selenoprotein S (Vimp)	1.02	1.18
**Other Superoxide Metabolism Genes:**
Arachidonate 12-lipoxygenase (alox12)	-1.50	1.48
Neutrophil cytosolic factor 1 (Ncf1)	1.21	1.32
Neutrophil cytosolic factor 2 (Ncf2)	-1.40	1.07
NADPH oxidase activator 1 (noxa1)	1.54	1.11
Si:dkey-48j7.2 (prex1)	1.46	1.45
Uncoupling protein 2 (ucp2)	1.05	-1.24
**Other Reactive Oxygen Species (ROS) Metabolism Genes:**
Aldehyde oxidase 1 (Aox6)	1.15	-1.07
BCL2/adenovirus E1B interacting protein 3 (bnip3)	1.14	1.06
Epoxide hydrolase 2, cytoplasmic (ephx2)	1.89	1.67
MpV17 transgene, murine homolog, glomerulosclerosis (mpv17)	-1.07	1.19
**Oxidative Stree Responsive Genes:**		
Apolipoprotein Ea (apoea)	1.31	-1.15
ATX1 antioxidant protein 1 homolog (yeast) (atox1)	1.17	1.03
BCL2-associated athanogene 2 (bag2)	-1.03	1.09
Cytoglobin 2 (cygb2)	1.03	1.02
24-dehydrocholesterol reductase (dhcr24)	-1.77	-1.50
Dual oxidase (duox)	-4.21	-2.14
Dual specificity phosphatase 1 (dusp1)	1.11	-1.36
Four and a half LIM domains 2a (fhl2a)	1.06	1.23
Forhead box M1 (foxm1)	1.18	1.04
Ferritin, heavy polypeptide 1a (fth1a)	1.25	-1.13
Glutamate-cysteine ligase, catalytic subunit (gclc)	1.21	1.07
Glutamate-cysteine ligase, modifier subunit (gclm)	1.05	1.03
Hexose-binding lectin 1 (hbl1)	1.24	-1.03
Heme oxygenase (decycleing) 1 (hmox1a)	1.12	1.20
Heat shock cognate 70-kd protein (hsp70)	1.60	2.04
Heat shock cognate 90-alpha 2 (hsp90aa1.2)	-1.20	1.40
Zgc:165670 (lhpp)	-1.41	-1.30
LOC101883157 (txnrd2)	1.02	1.53
NAD(P)H dehydrogenase, quinone 1 (nqo1)	1.41	1.22
Nudix (nucleoside diphosphate linked moiety X)-type motif 1 (nudt1)	-1.35	-1.14
Oxidative-stree responsive 1b (oxsr1b)	1.17	1.06
PDZ and LIM domain 1 (elfin) (pdlim1)	1.18	-1.03
Zgc:153084 (pnkp)	1.04	1.12
Prion protein (prnpa)	1.73	1.66
Prostaglandin reductase 1 (ptgr1)	1.78	1.21
Ring finger protein 7 (rnf7)	-1.01	-1.17
Si:dkey-217m5.5 (scara3)	1.07	1.52
Selenoprotein P, plasma, 1a (sepp1a)	1.34	-1.06
Sirtuin 2 (sirt2)	1.22	1.29
Sequestosome 1 (sqstm1)	1.01	1.33
Sulfiredoxin 1 homolog (S. cerevisiae) (srxn1)	1.23	-1.39
Serine/threonine kinase 25b (stk25b)	1.23	1.19
Zgc:92903 (txn)	-1.29	1.05
Thioredoxin reductase 1 (txnrd1)	1.21	1.20
**Oxygen transporters**		
Cytoglobin 2 (cygb2)	1.03	1.02
Myoglobin (Mb)	2.68	-3.02

**Table 3:** Top hits of oxidative stree related genes involved in RIC signaling by microarray analysis.

Gene symbol	Gene name	Function	Fold change	
			No wounding	1hour post wounding
DUOX	NOX/DUOX family of NADPH oxidase	generate H_2_O_2_	-4.21	-2.14
MB	myoglobin	faciliate oxygen transport, the storage of oxygen and a scavenger of nitric oxide or reactive oxygen species	2.68	-3.02
TINA	titin	myofilament protein, acts as a molecular spring in the cardiac sarcomere	1.28	2.22
HSP70.3	Heat shock cognate 70-kd protein, tandem duplicate 3	protect cells from heat or stress	1.59	2.04

NF-E2-related factor 2 (nrf2) is the master regulator of antioxidants, phase II detoxification enzymes and other cellular protective proteins through the antioxidant responsive elements (AREs) in the promoter of these genes. Among the best-characterized nrf2 downstream genes are those encoding NAD(P)H quinone oxidoreductase 1 (NQO1), heme oxygenase 1 (HO-1), prostaglandin reductase 1(PTGR1), thioredoxin reductase 1 (TXNRD1), glutathione synthetase (GSS) and glutamate-cysteine ligase (also known as γ-glutamylcysteine synthetase) modifier subunit (GCLM) and catalytic subunit (GCLC) [26]. In order to determine the role of nrf2 in RIC signaling, we examined the expression of genes involved in Nrf2-ARE pathway from the microarray data, which includes hmox1a, nqo1, ptgr1, gstk1, gss, ephx2, gclc, gclm. Interestingly, we found that the expression of hmox1a, nqo1, ptgr1, gstk1, gss, ephx2, gclc were all increased in RIC treated fish regardless of the tailfin condition, although these did not reach our defined 2-fold change (Table 4).

**Table 4:** Nrf2-downstream genes with ARE in their promoter are up-regulated by RIC.

Gene symbol	Gene name	Fold change	
		No wounding	1hour post wounding
HMOX1A	Heme oxygenase 1	1.12	1.20
NQO1	NAD(P)H dehydrogenase, quinone 1	1.41	1.22
PTGR1	Prostaglandin reductase 1	1.78	1.21
GSTK1	Glutathione S-transferase kappa 1	1.59	1.15
GSS	Glutathione synthetase	1.43	1.29
EPHX2	Epoxide hydrolase 2	1.89	1.67
GCLC	Glutamate-cysteine ligase, catalytic subunit	1.21	1.07
TXNRD1	Thioredoxin reductase 1	1.21	1.20

### RIC works through activation of Nrf2

**Fig 5:**
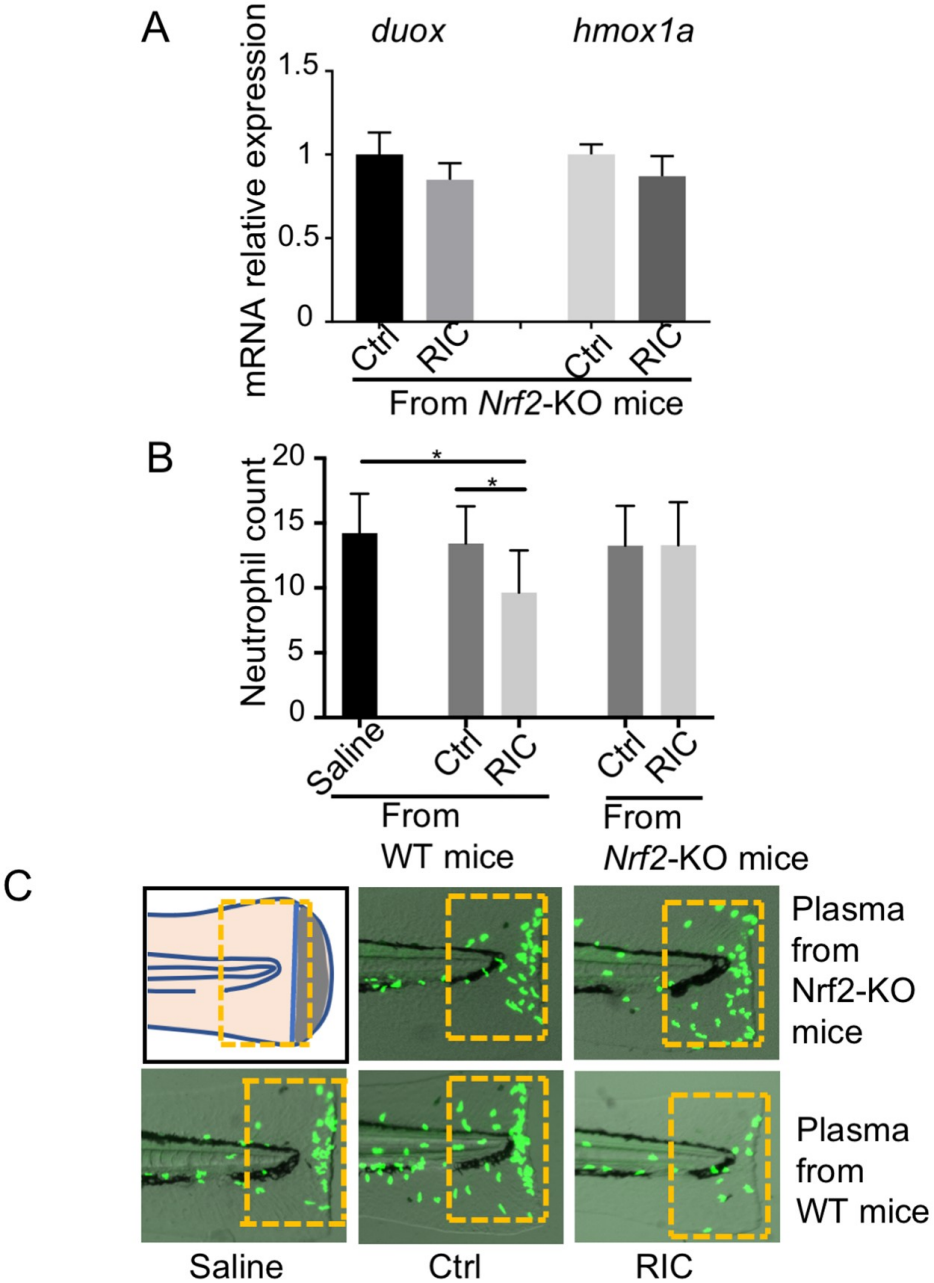
nrf2a plays a critical role on the protective effect of RIC. (A) Real time PCR showed the expression level of duox and hmox1a in the fish treated with the plasma derived from Nrf2-KO mice. (B) RIC plasma derived from Nrf2 KO animals did not exert inhibiting effect on neutrophil migration. (n = 28) *p<0.05. (C) Representative images of neutrophil migration from each group were shown. Yellow rectangular frames were used for quantitation.

In our previous work using a murine model of hemorrhagic shock, we showed that the nrf2-hmox1 was a key pathway in exerting the protective effect of RIC [27]. We therefore studied expression of the gene hmox1a in zebrafish using real-time RT-PCR. Fig 4B illustrates the effect of RIC plasma injection from wildtype mice into fish without (two bars on the left) and with tailfin cut (right two bars). As shown, under both conditions, there was an upregulation of hmox1a in RIC treated zebrafish (Fig 4B). By contrast, when RIC plasma was derived from nrf2 KO mice, the induced rise in hmox1a was not observed (Fig 5A). Similarly, when RIC plasma was derived from nrf2 KO mice, the induced rise in duox was not seen (Fig 5A). Finally, as shown in Fig 5B and 5C, the inhibitory effect of RIC plasma on neutrophil migration was reversed when RIC plasma was derived from nrf2 KO mice. Together, these point to the central role of nrf2 in mediating the effect of RIC injection.

**Fig 6:**
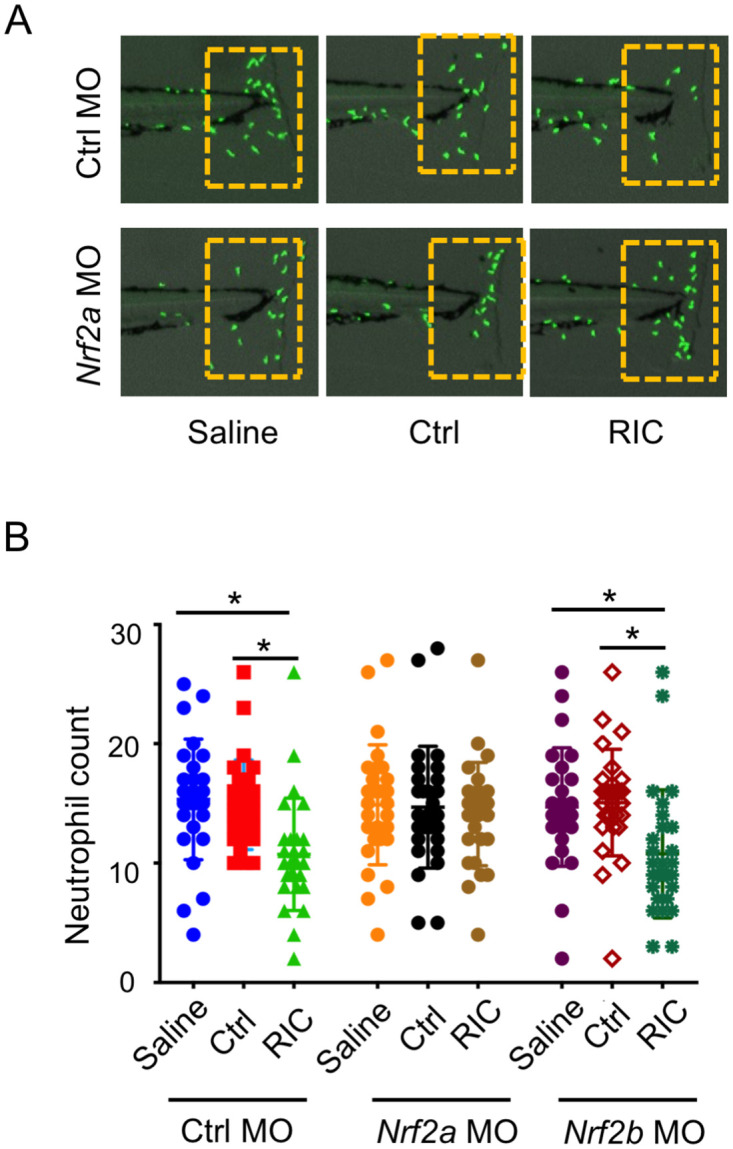
Knockdown of nrf2a abolished inhibiting effect of RIC on neutrophil migration. (A) RIC plasma shows significant inhibiting effect on neutrophil migration to the site of tailfin injury in MO-Ctrl and MO-nrf2b injected larvae, *p<0.05. However, there was no significant difference between these group in MO-nrf2a injected larvae. Representative images of neutrophils in the tailfin wound area were shown for each group. (B) Graph bars display knockdown of nrf2a by MO-nrf2a abolished inhibiting effect of RIC on neutrophil migration to the tail wound.

**Fig 7:**
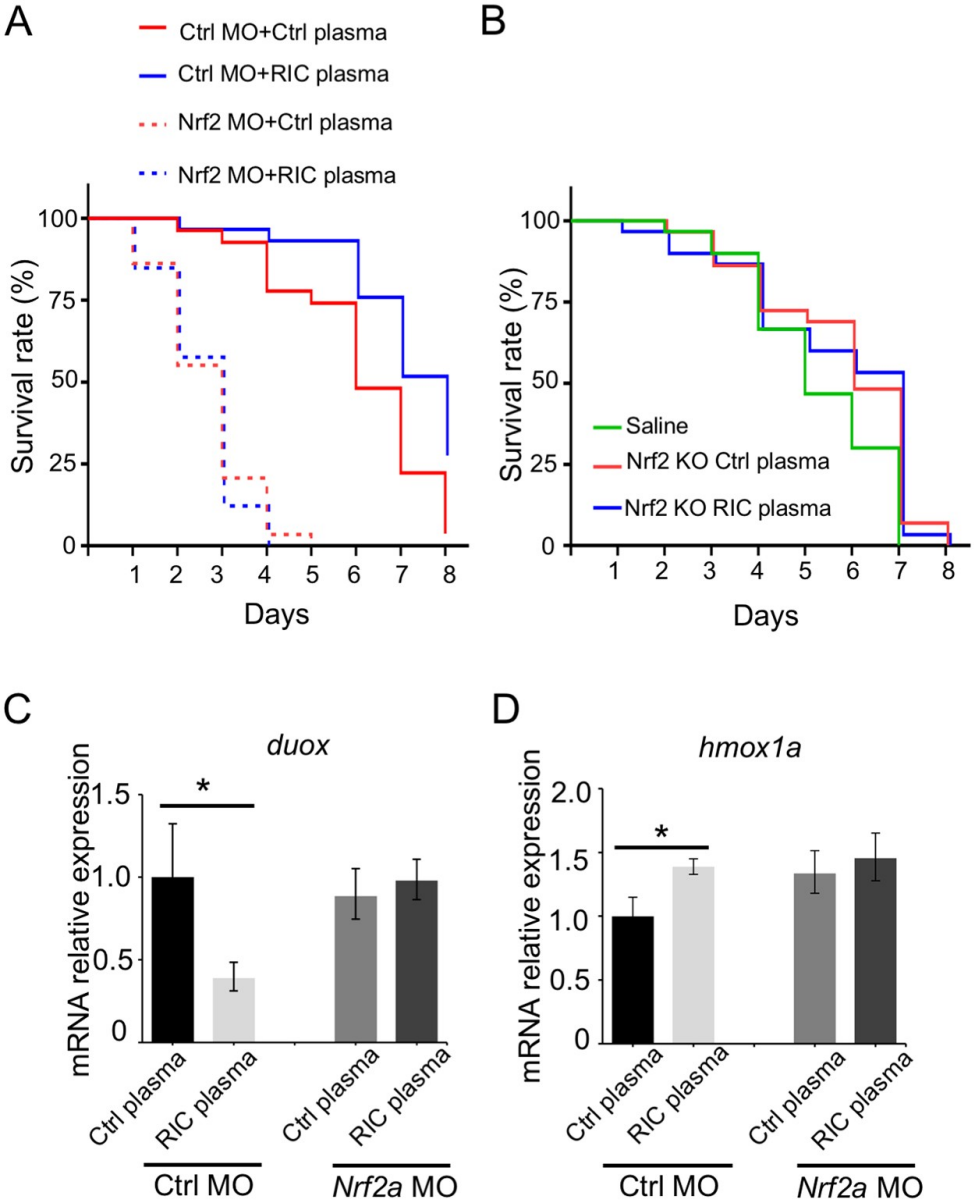
RIC works through activation of nrf2a in zebrafish. (A) Kaplan-Meier survival curve of zebrafish subjected to LPS treatment. nrf2a knockdown resulted in earlier mortality in response to LPS exposure (80 μg/mL) and the protective effect of RIC Plasma was abolished in nrf2a knockdown zebrafish (n = 26–33). (B) RIC Plasma from Nrf2 KO mice did not exert survival benefit in response to LPS exposure (80 μg/mL) induced mortality in zebrafish (n = 26–30). (C) Real time PCR showed the expression level of duox in the fish treated with the plasma in nrf2a-knockdown fish. *p<0.05. (D) Real time PCR showed the expression level of hmox1a in the fish treated with the plasma in nrf2a-knockdown fish. *p<0.05.

To further explore the role of zebrafish nrf2 function in the RIC-mediated anti-inflammatory effect, we generated morpholino knockdown of the two co-orthologs for human nrf2 in zebrafish: nrf2a and nrf2b [24]. As shown in Fig 6A and quantitated in Fig 6B, zebrafish injected with nrf2a-MO reversed the inhibitory effect of RIC on neutrophil migration to the site of injury, while no significant change was observed from zebrafish injected with nrf2b or control morpholino, implying nrf2a acts the role as a regulator for RIC, whereas nrf2b shows no obvious function. In addition, survival studies in animals exposed to LPS demonstrated that zebrafish injected with control morpholino were again protected by injection with RIC plasma (solid lines—P < 0.01 by Log-Rank test) (Fig 7A). However, knockdown of nrf2a resulted in earlier mortality when compared to zebrafish injected with control morpholino and exposed to exogenous H2O2 (dotted lines). In addition, RIC plasma derived from Nrf2 KO mice did not exert protection in WT zebrafish (Fig 7B). Finally, we examined the gene expression of duox and hmox1a in morpholino zebrafish treated with the plasma derived control or RIC mice (Fig 7C). Real-time PCR showed that the upregulation of hmox1a and downregulation of duox in presence of RIC plasma (left bars in Fig 7C and 7D) were not observed in Nrf2a morpholino zebrafish (right bars in Fig 7C and 7D). Collectively, these findings provide further evidence that the effect of RIC plasma involves the nrf2 pathway.

## Discussion

The present study provides novel insights into the mechanisms underlying the organ protective effects of RIC by examining how plasma derived from mice undergoing RIC exerts anti-inflammatory effects in zebrafish. The data show that ROS generation at the site of tailfin cut injury, a known chemoattractant for neutrophils [20], is significantly reduced by prior injection with RIC plasma and correlates with reduced neutrophil migration to the wound area. Further, RIC plasma exerted protective effects in two distinct models known to induce injury by oxidative stress, namely exposure to LPS or H2O2, where mortality was reduced, and the magnitude of tailfin injury was lessened. Together, these finding suggest that induction of an antioxidant response in zebrafish larvae by RIC plasma is an important contributor to its protective anti-inflammatory effect.

The master transcription regulator of antioxidative response, nrf2, appears to play a critical role in the protective effects of RIC. In zebrafish, there are two paralogs, displaying distinct functions which are co-orthologous to the human NRF2. The zebrafish nrf2a, located on chromosome 9, is similar to the position of the human NRF2 gene adjacent to the HOXD cluster. However, Nrf2b is more divergent than Nrf2a. It is located on chromosome 6, near miR-10d2 and other region corresponding to the location of the degenerate hoxdb cluster. Nrf2a and Nrf2b share 25.1% amino acid sequence identity overall, with greater identity found in the conserved 6 Neh (Nrf2 ECH homology) domains. The expression pattern of nrf2a and nrf2b are distinct during zebrafish embryonic development and in adult tissue. Most notably, nrf2a is consistently higher that of nrf2b among adult tissues, whereas nrf2b is 10–100 fold higher than that of nrf2a through developmental stages. In zebrafish, nrf2a acts mainly as an activator as a transcription factor, whereas nrf2b plays a role as a repressor [24]. Supporting a role for nrf2a, the studies show that the protective effect of RIC plasma was lost in nrf2a MO morphants but remained in the nrf2b MO morphants. This differential effect is consistent with the work of Timme-Laragy and colleagues [24] who demonstrated that the nrf2b paralogs of the nrf2 gene did not exhibit protection against oxidative stress compared to nrf2a. Further evidence for the role of nrf2 is seen in the studies of gene expression. The rise in the nrf2 dependent gene hmox1, known to be involved in the RIC plasma-mediated protection [27] did not occur in nrf2a MO morphants. In addition, the oxidant-generating gene duox, known to be a generator of oxidative stress and critical to neutrophil migration in the zebrafish tailfin cut model, was reduced by exposure to RIC plasma. This would be in keeping with the ability of RIC plasma to lessen neutrophil migration to the tailfin cut. Interestingly, in the nrf2a morpholino injected morphants, the reduction in duox did not occur (see Fig 7C), consistent with a role for nrf2 in the RIC-induced changes in duox expression. The mechanism whereby nrf2 regulates duox expression is not known and there are no data reporting direct regulation of duox by nrf2. Alternatively, nrf2 might regulate duox indirectly. Pacquelet et al. found an inhibitory function of NoxA1on DUOX activity in airway cells. The activity of mature DUOX is regulated by Ca2+ concentration through triggered dissociation of NOXA1. Our anti-oxidant array revealed an upregulation of noxa1 of 1.54- fold (without tailfin cut) and 1.11-fold (with tailfin cut) in zebrafish with RIC compared to control groups, suggesting that RIC might downregulate duox through upregulation of noxa1. Binding sites for Nrf2 are located in the promoter region of Nox4 [28]. However, further investigation appears warranted to elucidate the details.

The oxidative stress PCR array revealed several other potential candidate molecules potential contributing to the protective effect of RIC. For example, there was an upregulation of hsp70 (2.04- fold up) in RIC treated fish. Previous reports have suggested a cyto-protective effect of hsp70 following I/R in mice and cell cultures. Heat shock protein 70 (Hsp70) is a stress-responsive cellular protein and provides stress tolerance and cytoprotection against damage under ischemic conditions [29, 30]. Induction of Hsp70 either by enforced overexpression with viral vectors or pharmacological approaches protects brain and renal cells from ischemic injuries [29, 31–33]. Induction of Hsp70 contributes to the cardio-protection in several types of pre-conditioning and post-conditioning, thus it is considered as a promising therapeutic target for myocardial I/R injury [34]. Hsp70 protects cells by chaperoning damaged proteins and restoring their functionality in normal folded state, enhancing the activities of two main antioxidative enzymes glutathione peroxidase (GPx) and glutathione reductase (GR), and by inhibiting apoptosis [35, 36]. Our observation that zebrafish injected with RIC plasma expresses higher level of hsp70 compared to control plasma injected fish is in line with the protective effect of RIC. It indicates that the protection of RIC may partly attributed to the upregulation of hsp70, which enhances the ability to tolerate and remain redox homeostasis in response to I/R stress. Raghunath et al. have identified functional antioxidative responsive elements (AREs) in the promoter of Hsp70 in mice and zebrafish, which implies that Hsp70 is upregulated by Nrf2 /keap1-ARE pathway [37]. The upregulation of hsp70 probably resulted from the activation of ARE in its promoter through enhanced activity of nrf2a by RIC, which provides another piece of evidence to confirm that RIC leads to activation of nrf2a.

We also found many key antioxidants were upregulated by RIC, including nqo1, hmox1a, txnrd1, gstks, gclc, gclm, gss, ephx2, ptgr1, titin. Among them, gstks, gclc, gclm, gss,nqo1 and txnrd1 are enzymes involved in synthesis of glutathione (GSH) and thioredoxin (TXN), which are the two main pathways for elimination of excess ROS [38, 39]. nqo1 and hmox1a are two typical well-identified Keap1/Nrf2/ARE downstream genes in the maintenance of cellular redox homeostasis [40–42]. EPHX2, a soluble epoxide hydrolase (EH), is characterized to play diverse roles in xenobiotic metabolism. The upregulation of these genes by RIC indicates that RIC has important implications for potential antioxidant strategies that modulate levels of ROS.

In particular, DUOX is a protein belonging to the NOX family of ROS-generating NADPH oxidases. It is noteworthy that the studies by Niethammer and colleagues showed that duox is the major hydrogen peroxide producer at the site of tail wound injury and mediates neutrophil migration as a chemoattractant [20]. Our PCR array data showed that duox is down-regulated over 4.2-fold and 2.1-fold after RIC treatment in zebrafish without tailfin cut and with tailfin cut conditions, respectively. Real-time PCR further confirmed its downregulation in Fig 7. The observed downregulation of duox, the hydrogen peroxide producer, suggests a mechanism may potentially mediate the reduction of ROS generation for the RIC treated fish larvae at the molecular level.

There are a number of humoral factors shown to be involved in RIC, such as adenosine [43], bradykinin-2 [44], opioids [45], erythropoietin [46], and angiotensin-1 and prostaglandin [47] receptors. Work by Shimizu et al. [48], Breivik et al. [49] have also identified some hydrophobic humoral factors. While this suggests that a wide range of potential effector molecules in the plasma may be contributory, our study focuses on the Nrf2-antioxidant-ROS axis and demonstrates that Nrf2 activation is required for the RIC-mediated cyto-protection, suggesting RIC plasma may contain several molecules targeting Nrf2-antioxidant activities. The precise mechanism whereby RIC plasma activates the nrf2-dependent pathway was not studied in the current work. Nrf2/Keap1 are the cellular sensors of endogenous oxidative and electrophilic stress and plays the central role in the defense against cellular stress, forming a concerted machinery for cytoprotection and homeostasis. Typically, under basal conditions, Nrf2 stays in the cytoplasm with repressed anti-oxidative response due to the covalently binding to cysteine residues on its native repressor Keap1. Under conditions of electrophilic or oxidative stress, cysteine residues on Keap1 are modified, resulting in the translocation of Nrf2 into the nucleus, where it binds to the ARE in the promoter region of downstream gene and initiate the transcription of various cytoprotective enzymes. Yang et al recently reported that ischemic preconditioning in the brain elaborated endogenous lipid electrophiles, including 4-hydroxy-2-nonenal, which protected the mice against stroke through the activation of nrf2 [50]. Given that RIC is a form of ischemia/reperfusion injury, it is likely that the RIC induced sub-toxic ROS or other electrophile in the donor mouse plasma which were transferred to the zebrafish by injection, therefore activating nrf2. Clearly, further investigation is warranted.

Nrf2a is globally expressed in many different tissues including brain, eye, gill, gut, heart, kidney, liver, ovary, and testes from both male and female adult zebrafish [24]. However, it is unclear in which cells/tissues Nrf2/nrf2a is active to affect the oxidative stress response following injury. Kong et al. reported Nrf2 activation in myeloid cells alleviates inflammation in murine models. Deletion of Nrf2 in myeloid cells (neutrophils and macrophages) augments mortality in polymicrobial sepsis [51]. Nrf2 activity in macrophages and neutrophils is crucial in controlling lethal systemic inflammation. It is likely that nrf2a also acts as a critical immunomodulator in myeloid cells such as neutrophils and macrophages, control inflammatory response and protects against injury in zebrafish. More functional studies are warranted to elucidate the identity of the cells where nrf2a and its downstream genes play the main role to affect the oxidative stress response following injury.

We found it surprising that plasma derived from nrf2 knockout animals was unable to exert the protective effect in wild type zebrafish. Clearly, nrf2 is required for the elaboration of the “protective factor” into the plasma of mice undergoing the RIC procedure. Since the RIC procedure itself consists of repeated cycles of ischemic/reperfusion in the hindlimb, we speculate that this stress might induce a nrf2-dependent antioxidant response in the leg muscle and therefore, in the absence of nrf2, the response would likely be reduced and the constituents of the RIC plasma would be altered. Whether this reduces the elaboration of the protective humoral factor or results in the release of molecule(s) that counteract the protective effect is unclear. It is interesting, however, that these finding suggest that nrf2 plays a role in the afferent limb of the RIC effect (i.e. generation of the protective factor) and also in the effect limb of the RIC effect (i.e protection in the end organ).

## Conclusions

**Fig 8:**
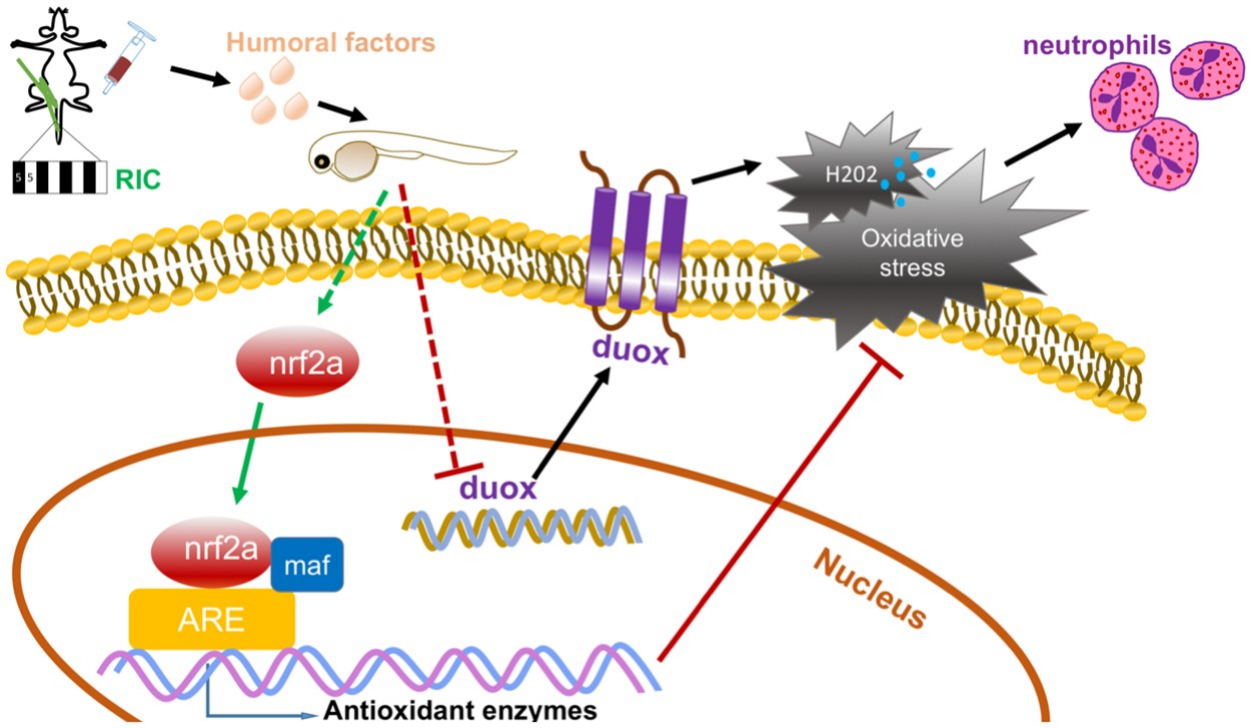
Graphical illustration of the protective effect of RIC in zebrafish.

Considered together, the results presented here suggest that the maintenance of a proper redox balance, as exerted by the humoral factors from RIC, is critical to its ability to the improved and reduced inflammation following injury. The humoral factors from RIC may upregulate antioxidative responses through activation of nrf2a and reduce ROS generation, partly by downregulation of duox, consequently leading to inhibition of neutrophil migration to the site of injury (Fig 8). Ultimately, RIC helps to maintain redox homeostasis in the site of injury and to retain balance of inflammatory response and innate immunity. Our findings provide us with additional evidence supporting the clinical usage of RIC in trauma patients.

## Supporting information

S1 Fig RIC plasma reduced number of neutrophils migration to the site of the injury when incubated under 32°C. (TIF)

S2 Fig Neutrophils counts in the whole zebrafish don’t show difference between RIC and control groups. (TIF)

S3 Fig CellROX fluorescence was quantitated at the edge of the tailfin wound. (TIF)

S4 Fig RIC plasma at the concentration of 1:100 dilution in saline did not show inhibitory effect on neutrophil migration. (TIF)

S1 Table Oxidative PCR profile array Ct value data sheet. (XLSX)

S1 Checklist (PDF)
