# Supplementary figures and images for: Plasma obtained following murine hindlimb ischemic conditioning protects against oxidative stress in zebrafish models through activation of nrf2a and downregulation of duox
Source: PLoS One. 2021 Nov 24;16(11):e0260442. doi: 10.1371/journal.pone.0260442 (PMC8612579; doi:10.1371/journal.pone.0260442)

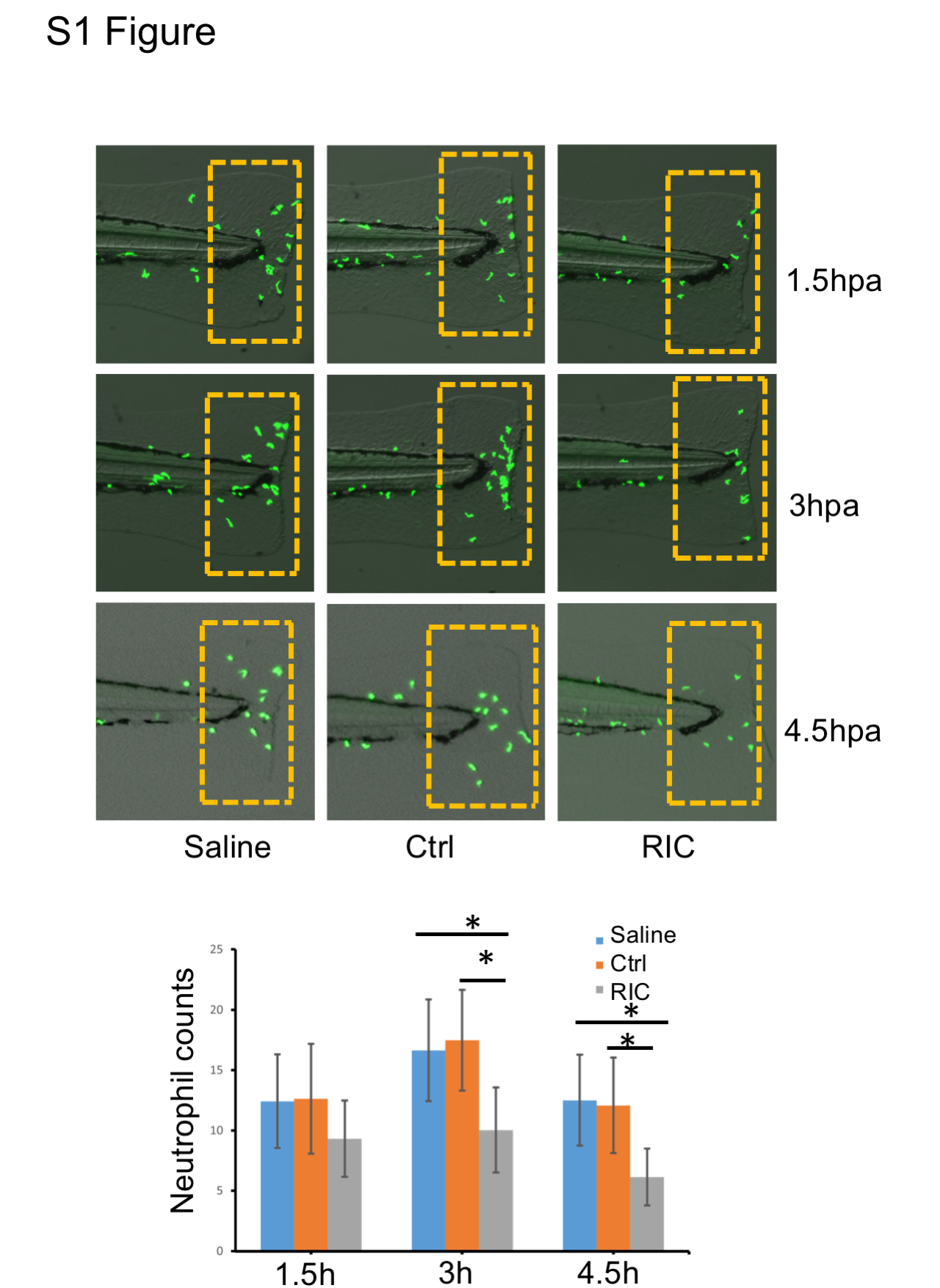

Supplement: S1 Fig — (TIF) [file pone.0260442.s001.tif]

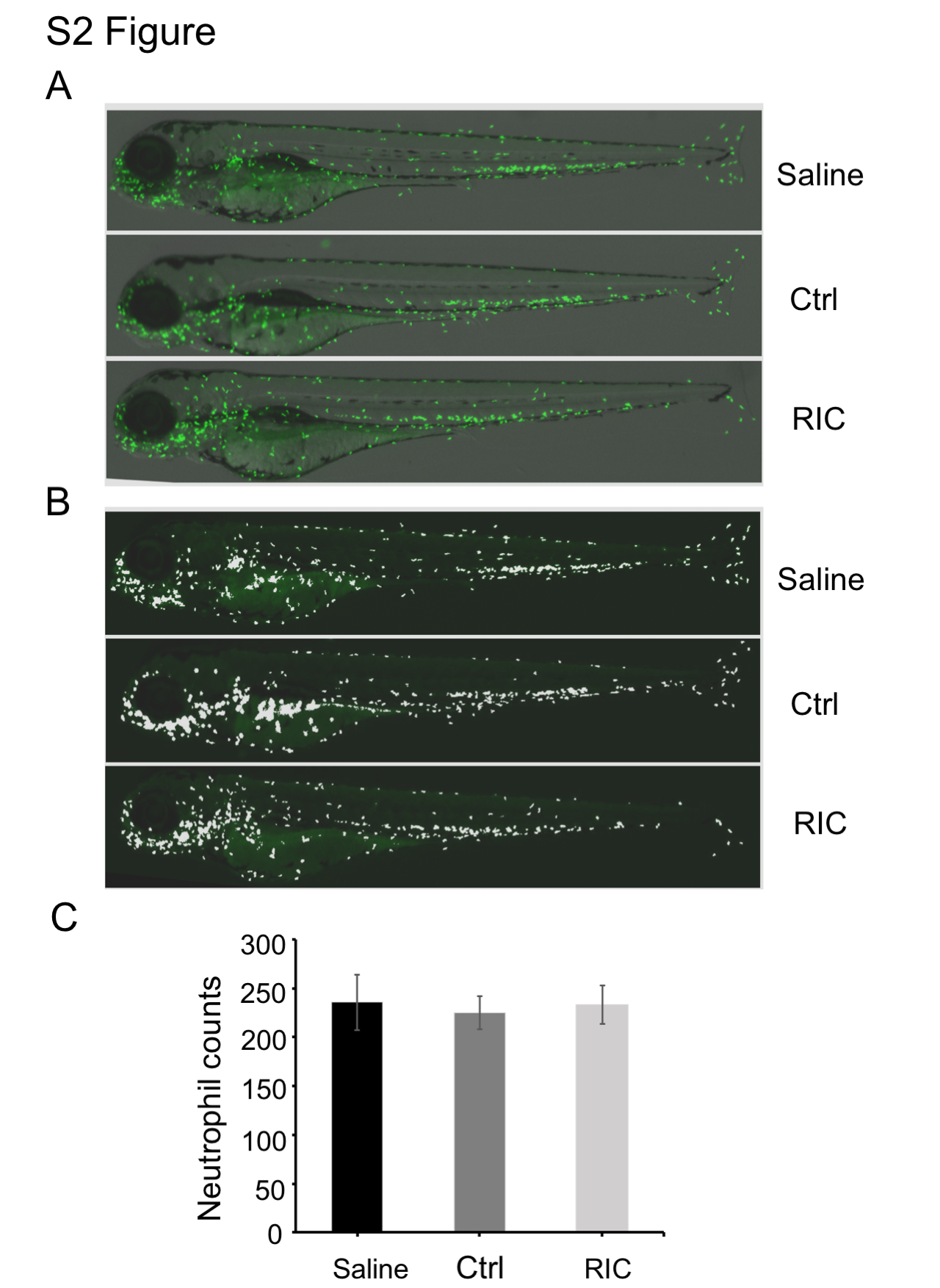

Supplement: S2 Fig — (TIF) [file pone.0260442.s002.tif]

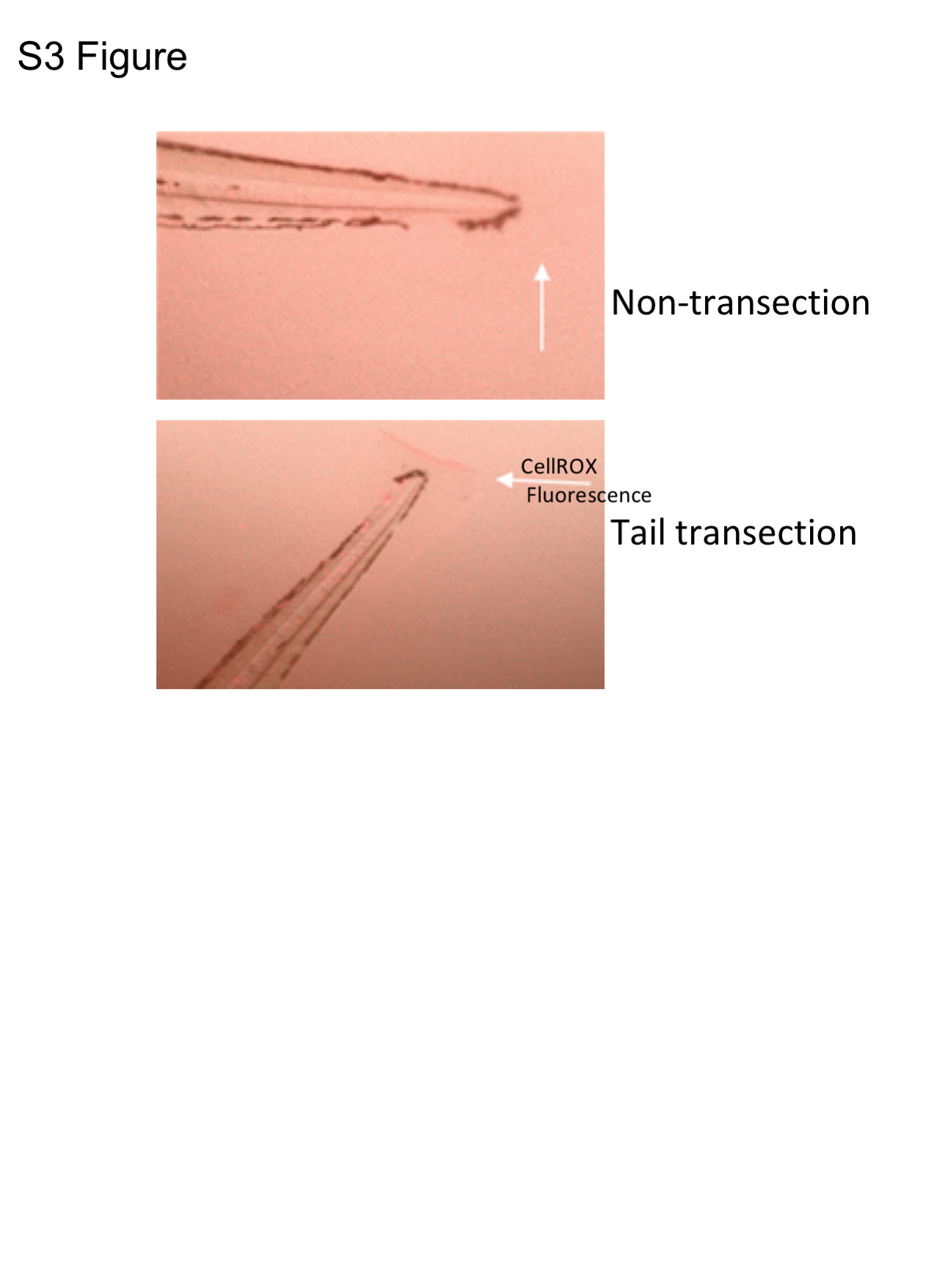

Supplement: S3 Fig — (TIF) [file pone.0260442.s003.tif]

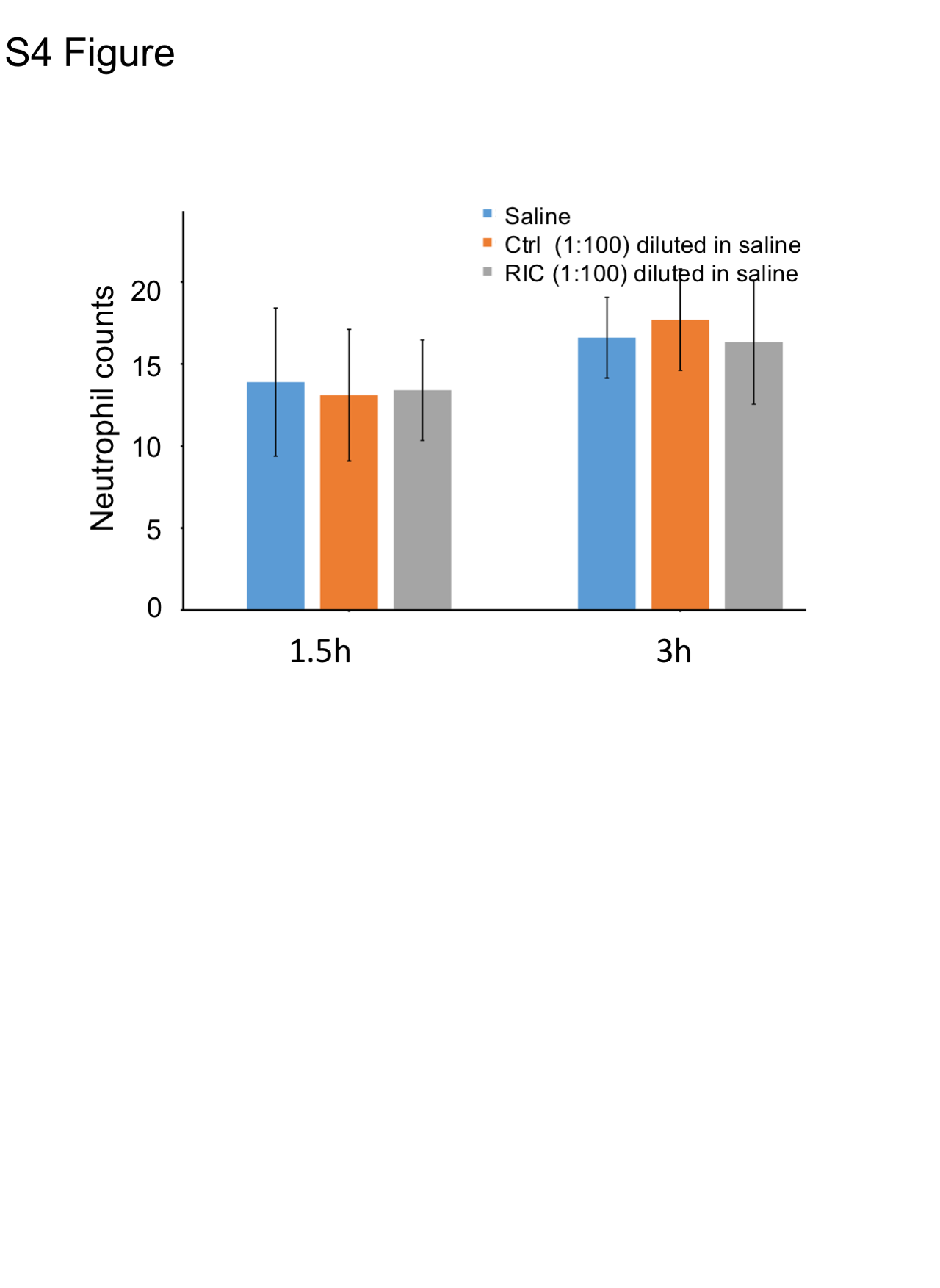

Supplement: S4 Fig — (TIF) [file pone.0260442.s004.tif]
